# Cell Line Derived Xenograft Mouse Models Are a Suitable *in vivo* Model for Studying Tumor Budding in Colorectal Cancer

**DOI:** 10.3389/fmed.2019.00139

**Published:** 2019-06-27

**Authors:** Laurent M. C. Georges, Olivier De Wever, José A. Galván, Heather Dawson, Alessandro Lugli, Pieter Demetter, Inti Zlobec

**Affiliations:** ^1^Department of Pathology, Hôpital Erasme, Université Libre de Bruxelles, Brussels, Belgium; ^2^Laboratory of Experimental Cancer Research, Ghent University, Ghent, Belgium; ^3^Institute of Pathology, University of Bern, Bern, Switzerland

**Keywords:** colorectal cancer, tumor budding, mouse model, xenograft, cell line, epithelial-mesenchymal transition

## Abstract

Tumor budding (TB) is an important prognostic parameter in colorectal cancer (CRC) and associated with metastasis. However, the mechanisms of TB have not been fully elucidated and a major limitation is the absence of *in vivo* models. Here, we determine the suitability of human cell line derived xenografts (CDX) as models of TB in CRC. Pan-cytokeratin (CK)-stained next-generation Tissue Microarrays (ngTMA) of two CDX models (HT-29, *n* = 12 and HCT-8, *n* = 8) and human CRC (*n* = 27 high-grade and 25 low-grade budding tumors, each) were evaluated for TB. Immunohistochemistry for E-cadherin, β-catenin, Ki-67, ZEB1, and TWIST1 was performed. HT-29 and HCT-8 were predominantly high-grade and no/low-grade TB tumors, respectively. TB counts in the tumor center (intratumoral budding, ITB) were significantly higher in HT-29 CDX tumors compared to human CRC (*p* = 0.0099). No difference was found in TB counts at the invasion front (peritumoral budding, PTB; *p*=0.07). ITB and PTB were strongly correlated (*r* = 0.438 and *r* = 0.62 in CDX and human CRC, respectively). Immunohistochemistry profiles were comparable in CDX and human CRC tissues. TB in the CDX mouse models is phenotypically similar to human CRCs and highlights comparable protein profiles. The HT-29 CDX could be a suitable model for the *in vivo* assessment of TB.

## Introduction

Tumor budding (TB) in colorectal cancer reflects the detachment of tumor cells from the main tumor mass and is defined by the presence of single tumor cells or small tumor cell clusters. Although typically described at the invasion front (peritumoral budding, PTB), TB can also be investigated within the tumor center (intratumoral budding, ITB). Studies have shown that both ITB and PTB can potentially be useful criteria in the clinical management of CRC patients (1–3). The “International Tumor Budding Consensus Conference” (ITBCC) presented guidelines for the evaluation of TB, which is useful in two clinical scenarios (4): First, the presence of high-grade PTB at the invasion front in endoscopically resected pT1 colorectal tumors is positively associated with lymph node metastasis. These patients are therefore recommended to undergo surgical resection. Second, high-grade PTB in patients with stage II colorectal cancers have unfavorable prognosis and are recommended for adjuvant chemotherapy. A third scenario currently under investigation is the presence of ITB in preoperative biopsies of patients with neo-adjuvant treated rectal cancer, which may help predict the presence of the presence of lymph node and distant metastases (5, 6).

TB may also reflect cancer cells in an hybrid state of epithelial-mesenchymal transition [EMT; (7)]. Immunohistochemical studies have indeed repeatedly shown both increased nuclear β-catenin and reduced E-cadherin levels in the TBs compared to the tumor center. Others report the expression of EMT inducers such as ZEB1 and TWIST1 in surrounding fibroblasts, hence promoting the tumor budding phenotype. A major limitation to the understanding of the underlying mechanisms of TB is the absence of *in vivo* models. The aim of this study is to investigate whether CRC cell line derived xenograft mouse models (CDX) show TB that is phenotypically similar to human cancers and thus suitable as model for the study of TB in CRC.

## Methods and Materials

### Tissue

#### Human

Hematoxylin-eosin (HE) stained slides from 52 consecutive patients with resected primary CRC treated at the Hôpital Erasme in Brussels, Belgium (Université Libre de Bruxelles) between 2015 and 2017 were retrieved from the Department of Pathology. These slides were then pre-reviewed for TB and one representative slide per case was selected. Twenty-seven high-grade budding cases and 25 low-grade budding cases were identified. The corresponding formalin-fixed, paraffin-embedded tissue blocks were retrieved from the archives of the Department of Pathology. The use of this material was approved by the local ethics committee of the hospital (Ref. No. P2018/126).

#### Mouse

The study was conducted on 6-year old female immune-deficient Swiss nu/nu mice (Charles River Laboratories, l'Arbresle Cedex, France). The mice were injected with cancer cells from 4 different human CRC cell lines (HCT-8, HT-29, COLO320, RKO). The cell lines were purchased from the American Type Culture Collection (ATCC). The animal studies were approved by the Animal Ethics Committee of Ghent University, Belgium.

The cancer cell implantation was done by a surgical procedure under general anesthesia (IsoFlo, Abbott, Belgium) and analgesia (Ketoprofen, 5 mg/kg). After a small midline laparotomy, the caecum of the mouse was located and gently exteriorized. The tumor cells were injected into the caecal wall. The appearance of a small bleb marked the successful implantation. Finally, the caecum was carefully returned to the abdominal cavity and the laparotomy was closed. After implantation, tumor development was assessed weekly by bioluminescence imaging until 6 weeks after inoculation. The animals were sacrificed when signs of disease (rectal prolapse, obstruction of the large bowel and intussusception of the caecum) were observed. Necropsy was performed and organs were sampled for histological examination. Formalin-fixed, paraffin-embedded tissue blocks were generated from each CRC models and H&E slides examined. However, upon preliminary screening for tumor budding, only two models were selected for subsequent analysis: HT-29 and HCT-8, a high-grade and a low-grade budding model, respectively. Details regarding the models can be found in the papers of Tommelein and colleagues (8, 9).

### Immunohistochemistry Staining of Whole Slides

Each block was sectioned at 2.5 μm and immunohistochemistry (IHC) for cytokeratin (CK) was performed on an automated immunostainer (Leica Bond RX, Leica Biosystems). Pan-CK markers were used to stain epithelial cells. To avoid cross-reactivity, a mouse antibody was used on human samples (AE1AE3, Dako M3515, 1:200, DAB as brown) while a rabbit antibody was used for mouse tissues (Pancytokeratin, Novus Biological NB600-579, 1:200, DAB as brown). Then all samples were incubated with HRP (Horseradish Peroxidase)-polymer for 15 min and subsequently visualized using 3,3-Diaminobenzidine (DAB) as brown chromogen (Bond polymer refine detection, Leica Biosystems, Ref DS9800) for 10 min.

### Scanning and Next-Generation Tissue Microarray (ngTMA®) Construction

All CK-stained slides were scanned using a slide scanner (Pannoramic P250, 3DHistech, Hungary). Scans were uploaded onto a case-managing platform (Case Center, 3DHistech, Hungary). Each scan was then re-reviewed for the presence of TB and the following annotations of 1.0 mm in diameter were made using a digital TMA annotation tool:

- cases of low-grade budding: 3x tumor center with a blue annotation,
- cases of high-grade budding: 3x tumor center with a blue annotation and 3x budding areas at the invasion front with a red annotation

The tissue blocks were loaded into a semi-automated tissue microarrayer (TMA Grandmaster, 3DHistech, Hungary). Annotated digital scans were aligned with an image of the corresponding tissue block and punched out in the annotated regions, ensuring the capture of TB. Punched cores were transferred into a recipient paraffin block. This led to the construction of three ngTMAs; two containing human tissue, one with the low-grade budding tumors (75 spots) and one including the high-grade budding tumors (149 spots). The third TMA (84 spots) contained all mouse tissue.

### Immunohistochemistry Staining of the TMAs

All ngTMA blocks were sectioned at 2.5 μm and underwent HE staining and single IHC for CK as described above. The human and mouse ngTMAs were additionally stained for E-cadherin, β-catenin, Ki-67, ZEB1, TWIST1. Mouse antibodies were evaluated in parallel with the negative control in mouse tissue. This Negative control was the same mouse tissue, stained only with secondary antibody, to identify the cross-reactivity area and positive cells. Double-staining for Ku-80 and Vimentin was performed on the mouse ngTMA: In a first step, Ku-80, a nuclear marker specific for human cells was visualized using DAB (brown). In a second step, AP (Alkaline phosphatase)-polymer for 15 min, was used to identify the Vimentin and visualized using fast red as red chromogen (Red polymer refine Detection, Leica Biosystems, Ref DS9390).

Finally, the samples were counterstained with hematoxylin and mounted with Aquatex (Merck). All information on antibodies and protocols can be found in Supplemental Table 1.

### Evaluation

Each TMA core was evaluated for the presence of TB and manually counted according to the definition of Koelzer et al. (10). Namely, TBs were defined as single cells or small clusters of cells in the peritumoral stroma, in which the nucleus is clearly identifiable. Cytoplasmic pseudofragments, ruptured glands, mucin pools, and necrosis were excluded. According to the ITBCC guidelines, the size of tumor buds was limited to 4 cells (4). In addition to counts of CK-positive cells in the mouse TMA, expression of Ku-80 was evaluated, to ensure that structures identified as TBs were indeed of human origin. For all other markers, expression patterns were described based on visual assessment. Focus was placed on disrupted E-cadherin from the membrane, nuclear expression of beta-catenin, nuclear expression of Ki-67 in TB, as well as nuclear expression of ZEB1 and TWIST1 in both tumor buds and surrounding fibroblasts.

A detailed workflow is available in Georges (11).

### Statistical Analysis

Descriptive statistics were performed on TB counts in the tumor center and at the invasion front from all three arrays, including mean, median, minimum, and maximum scores. The difference in TB counts in human and mouse high-grade budding cases was analyzed using a non-parametric Wilcoxon Rank Sum test. Analyses were performed using SAS v9.2. *P*-values < 0.05 were considered statistically significant.

## Results

### Overview of Cases

**Figure 1:**
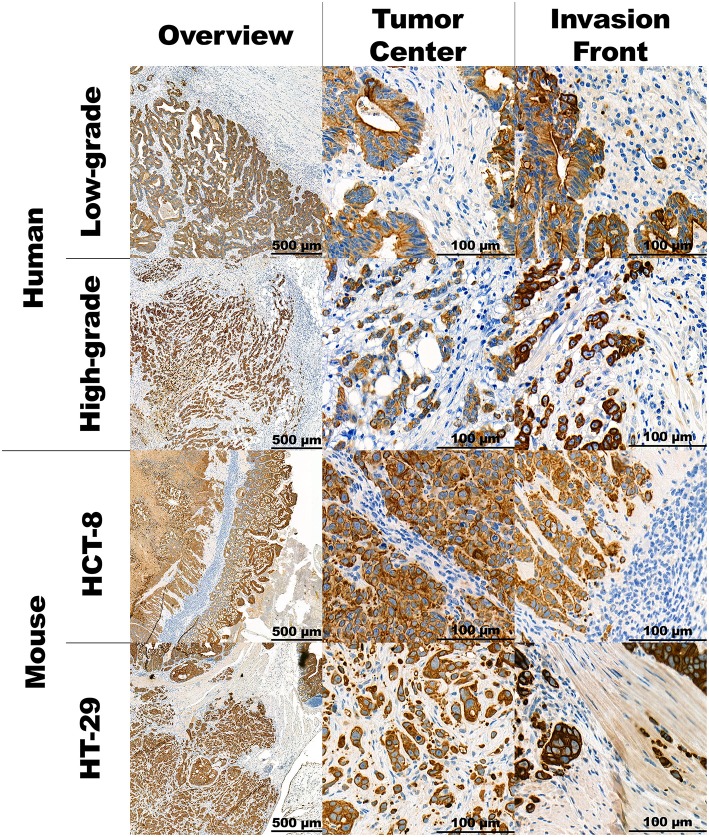
Panel with representative images of the human and mouse CRC stained in CK. Overview of the tumor at 5x magnification, the tumor's corresponding center and invasion front at 40x magnification.

**Figure 2:**
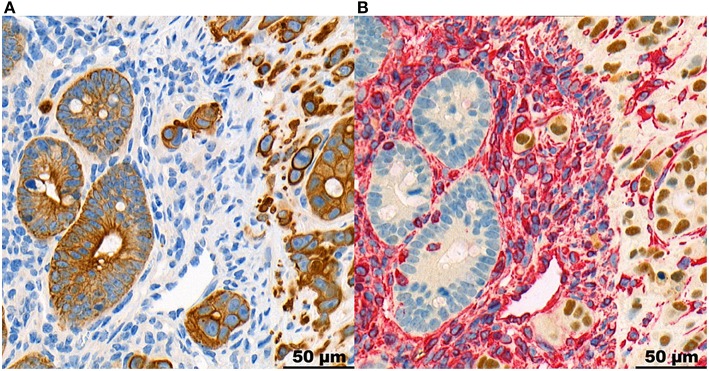
Panel with representative images of the same region at the invasion front of a mouse HT-29 tumor, stained in CK **(A)** and double-stained in Ku-80 and Vimentin **(B)**, at 40x magnification. It shows the tumor cells including TBs stained positive for Ku-80, proving their derivation from the human cell line. The tumor stroma is stained for vimentin in red. The normal intestinal mucosa of the mouse is negative for Ku-80.

Representative images of human and mouse tumors are found in Figure 1. Both the human high-grade budding tumors and the HT-29 CDX mouse models show single cells and small clusters of cells infiltrating the stroma. The definition of TB was easily applied to mice. The buds were very similar in size and shape. However, the human tumors showed a more spacious spread of TBs within the surrounding stroma. Positive staining for Ku-80 confirmed that CK-positive-appearing TBs in the mouse are in fact derived from the human cell line. Representative images can be found in Figure 2.

### Difference in TB Counts Between Human CRC and Mouse CDX Model

TB counts were evaluated in each TMA core. Descriptive statistics are found in Table 1. In total, 20 mouse tumors were analyzed, including 8 low-grade (HCT-8) and 12 high-grade budding tumors (HT-29). The mean number of buds per TMA core in the tumor center was 2.79 and 22.64, respectively. In the front, the average number of buds in the high-grade model was 45.0. The correlation between the count of TBs in the center (ITB) and the invasion front (PTB) was moderate for the HT-29 model (*r* = 0.438).

**Table 1:** Descriptive scores of TBs in human cancers and mouse xenograft models, in the tumor center and at the invasion front.

	**Budding status**	**n^**°**^ of cases**	**n^**°**^ of cores**	**Mean**	**Median**	**Minimum**	**Maximum**
**Tumor Center (n°** **of buds per core)**							
Mouse	Low (HCT-8)	8	19	2,79	1	0	11
	High (HT-29)	12	33	22,64	14	0	88
Human	Low	25	75	1,68	0	0	19
	High	25	75	11,96	3	0	121
**Invasion Front (n°** **of buds per core)**							
Mouse	Low (HCT-8)	8					
	High (HT-29)	12	29	45,00	47	4	91
Human	Low	25					
	High	25	74	30,31	22	0	156

*TB was not evaluated at the invasion front for the low-grade budding tumors*.

Similarly, in human cases, tissue of 50 patients was analyzed, including 25 low-grade and 25 high-grade budding cancers. The average budding counts per TMA core in the center were 1.68 and 11.96, respectively. At the invasion front of high-grade budding tumors, the average number of TBs per TMA core was 30.3. Again, ITB and PTB counts were highly correlated (*r* = 0.62).

Strikingly, the number of tumor buds within the tumor center of the high-grade budding tumors was significantly different between mouse and human, the former showing two times more budding counts than the human tissue (*p* = 0.0099). At the invasion front, in cores selected for high numbers of TBs, a non-significant difference was observed between mouse (45 buds on average) and human (30.3 buds on average; *p* = 0.07), despite the number of TBs still being markedly higher in the mouse.

### Immunohistochemistry Patterns in Human CRCs and Mouse CDX Models

**Figure 3:**
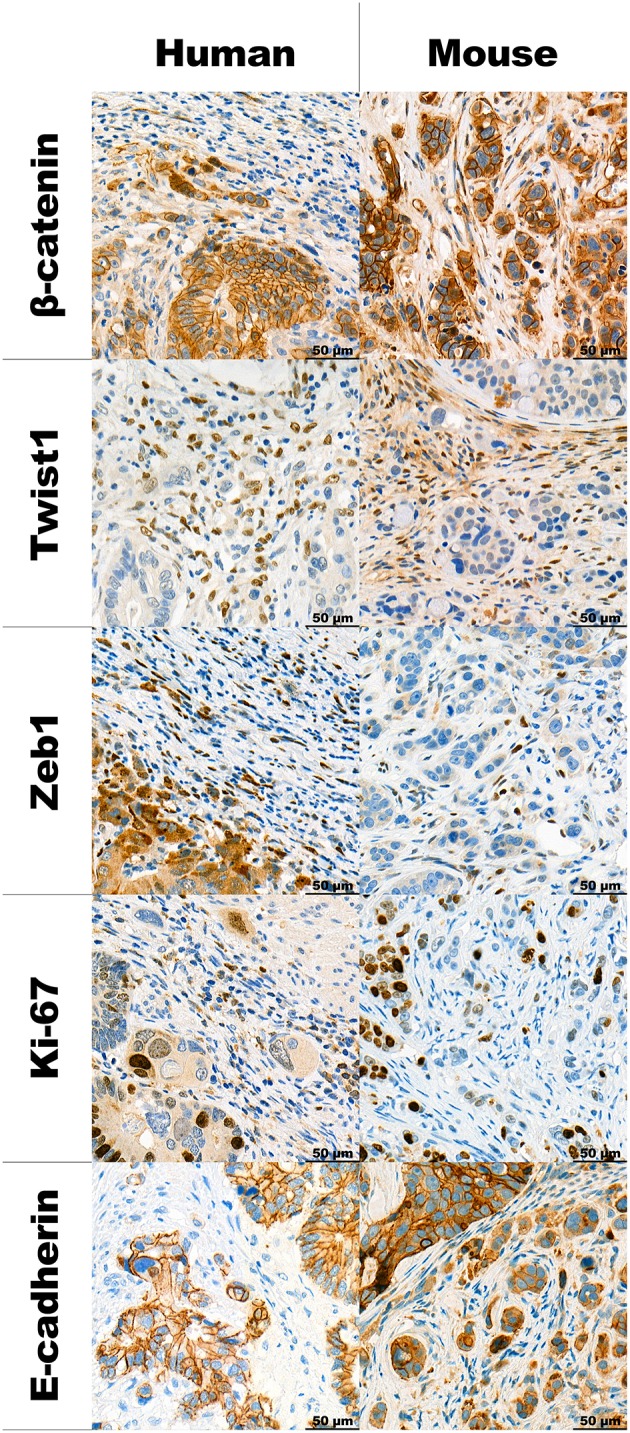
Panel with representative examples of TB in human high-grade budding tumors and HT-29 model, stained using different IHC markers at 40x magnification.

IHC stainings of the human and mouse tumors show that proteins, typically associated with EMT and budding are similarly expressed in both situations. The detailed description can be found in Table 2 along with representative images in Figure 3.

**Table 2:** Description of the expression of proteins, typically associated with EMT and Tumor Budding (TB), in the budding population in the human and mouse tumors.

	**Human TB**	**Mouse TB**
β-catenin	Predominantly membranous and cytoplasmic staining in the TBs Downregulation of membranous expression compared to the tumor center Rarely nuclear staining in TBs, but only when center also has nuclear staining	Predominantly membranous and cytoplasmic staining in the TBs Downregulation of membranous expression compared to the tumor center No nuclear staining of the TBs
Twist1	Nuclear staining of the mesenchymal cells	Abundant stromal positivity
Zeb1	Nuclear staining of the mesenchymal cells	Nuclear staining of the mesenchymal cells
Ki-67	Fewer cells of the TB population are positive, compared to the tumor center	Fewer cells of the TB population are positive, compared to the tumor center
E-cadherin	Downregulation of membranous expression in the TBs	Downregulation of membranous expression in the TBs

## Discussion

The novel findings of this study show that different orthotopic mouse xenograft models (HT-29 and HCT-8) can show a range of TB counts in both the center and the invasion front similar to what is seen in human tumors. Moreover, the immunohistochemistry pattern identified in human CRC tissues is concordant with that in mouse CDX models, suggesting that the latter may be appropriate for investigation of TB *in vivo*.

We used CK staining for evaluation of TB for several reasons: first, previous studies have reported 3-6x more TBs counted on CK in comparison to HE stains, second, the inter-observer agreement of budding in CK stains is markedly improved (12), third TBs can be camouflaged within the dense peritumoral stroma or inflammation, while on the other hand activated fibroblasts can be mistakenly identified as TBs (10). In a second step, we could confirm the presence of both PTB and ITB within the mouse tumors. Cells identified as TBs based on CK stains were also positive for Ku-80, confirming that budding cells were correctly interpreted as buds deriving from the human cell line.

Next, the correlation between ITB and PTB counts was evaluated. A strong positive correlation was observed in both human and mouse. It has previously been shown in human CRC, that budding within the main tumor mass is associated with budding at the invasion front (5). Our study shows that the same observation can be made in the orthotopic mouse xenograft model.

A subgroup analysis of high-grade budding tumors produced two important results. First, a significantly greater number of TBs was found within the center of mouse tissues, as compared to the center of human tissues. Although, it may be hypothesized that this result was due to the methodology of implanting cells into the caecum of the mice, the same observation of high numbers of buds in the low-grade budding mouse tissues should have been made but was not. This suggests a real biological phenomenon and not a consequence of the implantation method. Second, although higher numbers of TBs were found at the invasion front of the mouse tissues in comparison to the humans, the difference was not statistically significant.

We found similar immunohistochemistry expression profiles of the investigated proteins between human and mouse tissues. Previous laser capture microdissection studies in both CRC and oral squamous cell carcinomas show that tumor buds over-express EMT-related genes such as *ZEB1, ZEB2, DES, TGFB3*, and *VIM* in comparison to the main tumor mass (13, 14). We have also recently found that TB is significantly associated with the highly unfavorable CMS4 prognostic subgroup of the Consensus Molecular Subtypes, which is characterized as a “mesenchymal” subgroup of cancers overexpressing genes involved in EMT, TGF-beta activation, matrix re-modeling and WNT signaling (15, 16). We have also previously found that tumor buds show little Ki-67 staining, indicating their more non-proliferative nature (17). Results of this study are consistent with previous reports showing disrupted E-cadherin and infrequent Ki-67 staining. However, nuclear β-catenin staining was rare in TB and in our hands, was only found in cases where the main tumor also showed nuclear staining. Expression of ZEB1 and TWIST1 was found in the stromal cells in both human and mouse samples. This has previously been described in colorectal cancers and pancreatic ductal adenocarcinomas (18, 19). In fact, previous findings have shown strong correlations between TWIST1 positivity in fibroblasts and high-grade tumor budding areas. Interestingly, TWIST1 expression may be epigenetically regulated as shown by hypermethylation of the promoter region in stromal areas containing low /absent numbers of tumor buds.

Interesting is also that the two models showed such a range of budding counts, with the HT-29 being considered as a “high-grade” model and HCT-8 as a “non-/low-budding” one. Mice of both models lack an adaptive immune system. Here, we see both the presence and absence of TB in a tumor microenvironment without lymphocytes, suggesting that the underlying process of TB may be independent of the immune response. Nonetheless, reports suggest an inverse correlation between budding and the presence of peritumoral lymphocytic inflammation at the invasion front of cancers, particularly those with Microsatellite Instability (MSI) suggesting the immune system's reaction, and targeted destruction of TB (20).

We are not the first to look at a model of budding in an animal. Prall et al. took primary CRC from patients and transplanted these subcutaneously into mice. These patient-derived xenografts (PDX) also showed TB and podia formation similar to what was found in the primary diagnostic material (21). Although such an approach will allow the study of budding a personalized way *in vivo*, there may be several associated disadvantages, such as low success rates of the xenografting process and limited reproducibility. The approach presented in our current study used CDX models from two well-established cell lines and implantation of cells directly into the cecum. This has the additional advantage of studying TB in the target organ, namely the colon.

The use of digital pathology in this study had two major advantages. First, by evaluating digital scans of CK stained tumors, we could precisely annotate high-grade budding areas in multiple regions. These precise regions were captured and transferred into a ngTMA, which allows us to perform stainings to evaluate the same buds on serial cuts using different biomarkers. Serial alignment of images for cross-checking of tumor buds was possible using a scan viewer software. A limitation of this study is the annotation of areas of the tumor center which were not specifically selected to represent regions of budding. Nonetheless, since multiple regions of each tumor were annotated, we believe that the degree of ITB is accurately represented.

To summarize, this study shows that orthotopic mouse xenograft models from the human HT29 cell line and HCT-8 cell line are phenotypically similar to TB in human CRC, suggesting that these models can be further used to help elucidate the possible mechanisms behind TB in CRC.

## Ethics Statement

The protocol was approved by the Service de la recherche biomédicale, Hôpital Erasme. (Reference P2018/126).

## Author Contributions

PD, OD, IZ, and LG conceived and planned the study. OD provided the mouse materials. PD selected the human tissue samples. JG constructed the ngTMAs and carried out the immunostainings. LG carried out the scoring of the TBs in the human and mouse tissue. IZ contributed to the interpretation of the results. LG wrote the manuscript with the support of PD, IZ, HD, and AL. All authors provided critical feedback and helped shape the research, analysis, and manuscript.

### Conflict of Interest Statement

The authors declare that the research was conducted in the absence of any commercial or financial relationships that could be construed as a potential conflict of interest.
