# Supplementary material for: Cell Line Derived Xenograft Mouse Models Are a Suitable in vivo Model for Studying Tumor Budding in Colorectal Cancer
Source: Front Med (Lausanne). 2019 Jun 27;6:139. doi: 10.3389/fmed.2019.00139 (PMC6610335; doi:10.3389/fmed.2019.00139)
Supplement: Supplementary file 1 [file Table_1.docx]

**Supplementary Material**

**Supplemental Table 1:** List of antibodies used for immunohistochemical analysis.

| **Primary antibody** | **Tissue** | **Company** | **Ref. Number** | **Host** | **Dilution** | **Antigen retrieval** |
| --- | --- | --- | --- | --- | --- | --- |
| Pan-CK (AE1/AE3) | Human | Dako | M3515 | Mouse | 1:200 | Citrate buffer, 100° - 30 min |
| β-catenin | Human | Abcam | ab32572 | Mouse | 1:500 | Tris buffer,  95° - 30 min |
| E-cadherin | Human | Dako | M3612 | Mouse | 1:200 | Tris buffer,  95° - 30 min |
| Ki-67 (MIB1) | Human | Dako | M7240 | Mouse | 1:200 | Tris buffer,  95° - 30 min |
| Pan-CK | Mouse | Novus Biol. | NB600-579 | Rabbit | 1:200 | Tris buffer,  95° - 30 min |
| β-catenin | Mouse | Santa Cruz | sc-7199 | Rabbit | 1:200 | Citrate buffer, 100° - 30 min |
| E-cadherin | Mouse | Santa Cruz | sc-7870 | Rabbit | 1:200 | Citrate buffer, 100° - 30 min |
| Ki-67 (SP6) | Mouse | Thermo Scientific | MA5-14520 | Rabbit | 1:100 | Citrate buffer, 100° - 30 min |
| Twist1 | Human/Mouse | Abcam | Ab50887 | Mouse | 1:25 | Citrate buffer, 100° - 30 min |
| Zeb1 | Human/Mouse | Sigma Aldrich | HPA027524 | Rabbit | 1:200 | Citrate buffer, 100° - 30 min |
| Ku-80 | Human | CellSignaling | 2180 | Rabbit | 1:1000 | Tris buffer,  95° - 30 min |
| Vimentin | Mouse | Abcam | ab92547 | Rabbit | 1:2000 | Tris buffer,  95° - 30 min |
